# An Optimization Approach to Multi-Sensor Operation for Multi-Context Recognition

**DOI:** 10.3390/s21206862

**Published:** 2021-10-15

**Authors:** Raslan Kain, Hazem Hajj

**Affiliations:** Department of Electrical and Computer Engineering, American University of Beirut, Beirut 1107 2020, Lebanon; hh63@aub.edu.lb

**Keywords:** energy efficiency, Mobile Sensing, context-awareness

## Abstract

Mobile devices and sensors have limited battery lifespans, limiting their feasibility for context recognition applications. As a result, there is a need to provide mechanisms for energy-efficient operation of sensors in settings where multiple contexts are monitored simultaneously. Past methods for efficient sensing operation have been hierarchical by first selecting the sensors with the least energy consumption, and then devising individual sensing schedules that trade-off energy and delays. The main limitation of the hierarchical approach is that it does not consider the combined impact of sensor scheduling and sensor selection. We aimed at addressing this limitation by considering the problem holistically and devising an optimization formulation that can simultaneously select the group of sensors while also considering the impact of their triggering schedule. The optimization solution is framed as a Viterbi algorithm that includes mathematical representations for multi-sensor reward functions and modeling of user behavior. Experiment results showed an average improvement of 31% compared to a hierarchical approach.

## 1. Introduction

The advances in pervasive computing, such as smart wearables and sensor networks, have provided opportunities for health monitoring and human-centered context-aware systems. Mobile devices with sensory capabilities are commonly used to recognize the contexts of the user and provide appropriate assistance and services. Context relates to numerous areas of human-centric activities, such as health-care monitoring [1,2], activity recognition [3], social networking [4], location [5,6], and emotion recognition [7,8]. A context state describes one of many possible depictions of an entity within a context. States from separate contexts are mutually disjoint; for example, in the context activity, the user can be walking, sitting, or running. On the other hand, in the context of emotion, the states can be happy, sad, or angry. Context states are typically recognized by processing data collected from smartphone sensors (accelerometer, gyroscopes, GPS, etc.), wearable sensors (electrocardiograms (ECG), heart rate sensors, or body temperature sensors), or wearable devices (smartwatches and headsets).

A key issue with context-aware applications is the large demand on battery energy attributed to sensors’ power consumption [9], and algorithms that increase computational workload [10]. To minimize delays in context recognition, sensors would need to operate continuously, which in turn would cause larger energy consumption. It would be more energy-efficient to use the sensors intermittently, by turning them off when a state remains unchanged and then back on when a new state is expected to be encountered. Unfortunately, the time of state change cannot be perfectly predicted beforehand, since anticipation of state changes is not deterministic and is still a challenge in the field of anticipatory mobile computing [11]. As a result, the sensor operation problem can be formulated as an optimization problem that tries to balance between minimal delays in the detection of change and energy consumption while taking into account the expected timing of a new context change.

Furthermore, with the proliferation of context recognition applications, there is an opportunity for synergy across choices of sensors when multiple contexts are operating. In such cases, the choice of sensors should minimize the energy consumption for all contexts simultaneously, not just for each context separately.

There has been active research to devise efficient sensing mechanisms for context recognition. Some methods work for individual sensors or single contexts [12,13], while focusing on sensor selection and sensor scheduling. Other methods [14,15,16] consider the trade-off in energy, accuracy, and delays for multiple groups of sensors and multiple contexts. The proposed hierarchical approaches first select groups of sensors and then determine individual sensing schedules. Their main limitation is their lack of accounting for potential synergy across contexts, which results in impacts on sensor selection and scheduling.

The aim of this work was to address this limitation by proposing a holistic optimization approach that can simultaneously consider sensor selection and sensing schedules towards the optimal trade-off between overall energy consumption and delays in detecting desired contexts. The solution is framed as a Viterbi algorithm with personalized capture of user behavior that reflects the most probable instances for change in context. The Viterbi algorithm operates offline to generate sensing schedules, modeled according to personalized user behavior, for sensor groups categorized by the associated context recognition model. After that, the sensors are selected in real-time by solving a multi-objective optimization problem considering the energy and delay components of the combined operation, following the related sensing schedules of different sensor combinations. Moreover, we take into consideration the synergistic effects of different sensor combinations by taking advantage of sensors used in different context recognition models. The contributions of this work are:

- A holistic optimization formulation for simultaneous decisions on both sensor choices and sensor schedules as opposed to a hierarchical optimization scheme.
- The definition of holistic reward functions for sensor scheduling that account for energy, accuracy, and delay for the complete set of contexts simultaneously.
- A user behavioral model that estimates the probability of context state change customized to the user’s behavioral pattern.

The rest of the paper is organized as follows: Section 2 reviews related work in the literature for energy efficiency techniques. Section 3 describes the optimization problem and the mathematical formulation, and the notation used. Section 4 presents an overview of the proposed system that solves the optimization problem. Section 5 showcases the experiments conducted to evaluate the proposed solution in comparison to the state-of-the-art. Section 6 presents and discuss the practical aspects of our solution approach. Finally, Section 7 presents the conclusion.

## 2. Literature Review

Related previous work on efficient sensors’ operations for context recognition can be categorized into work that has considered sensor selection only, sensor scheduling, or both.

### 2.1. Sensor Selection

Taleb et al. [17] presented an algorithm that uses a heuristic that consists of choosing the sensors that maximize the ratio of the accuracy divided by the energy consumption with constrains based on sensor availability and battery level. Gao et al. in [18] proposed a framework that selects a set of sensors to reduce energy consumption attributed by selecting a subset of the initial sensors based on the context state and relying on expert knowledge. Convex optimization is used to minimize a trade-off between transmission energy and the probability context misrecognition. A framework presented by Kang et al. [19], called SeeMon, selects a set of sensors named the essential sensor set (ESS) by solving a variation of the minimum set cover problem by greedily selecting the most cost-effective sensors iteratively capable of recognizing the context while trading-off computational complexity and energy savings in terms of data transmission rate. The ESS updates either continuously or periodically based on the available battery levels.

Dynamic sensor selection, by Zappi et al. [20], trades-off power consumption with recognition accuracy by adapting the set of sensors once the energy of sensor nodes (smart-phone, smart-watch, etc.) depletes. The approach either selects the sensor cluster that gives the highest accuracy after enumerating all the possibilities, or selects the sensor cluster that first meets an accuracy threshold during enumeration. Another approach by Gordon et al. [21] selects sensors based on the predicted future activity state of the user using a first-order Markov chain. The approach evaluates a weighted mapping of each activity to the sensors in terms of the loss in accuracy compared to other sensors. The weighted map is generated using a nearest neighbor classifier for all training vectors for each state and simulating different feature combinations, where each feature combination is linked to a sensor group, and then seeing the varying effect on the accuracy of different combinations.

In a recent approach, Janko et al. [13] selected a sensor and their sampling frequency through an iterative approach with a multi-objective optimization for a trade-off between energy and accuracy. The optimization problem is solved using a genetic algorithm (NSGA-II). The sensors and their settings are specified according to the currently recognized context state and the most likely up-coming state, assuming the Markov property holds. Moreover, the same authors introduced in [22] a cost-sensitive decision tree to detect context states with finer granularity in terms of feature data, e.g., running fast vs. running slow, as each one could have a different optimal choice of sensors. Each node of the tree is a pair of a context classifier and set of sensors, as such, the method traverses the tree from the node (default setting) until a leaf is reached that yields the highest value calculated as the accurate classification minus a weighted energy cost. Jaimes et al. [12] proposed a method that cycles through different sensors and weighs the information with each sensor feature versus the energy cost of using the sensor. The drawback of the approach is that it cannot handle quick changes in context state and is prone to delays due to the cycling mechanism. Starliper et al. [23] presented an approach where power consuming physiological response sensors are activated or deactivated depending on the activity recognized.

### 2.2. Sensor Scheduling

Some approaches aim to reduce energy consumption not by scheduling sensor operation, but by scheduling data communications to reduce energy consumption and delay. Such approaches, [24,25] for example, use a smart-update policy in which sensor data is continuously collected and communicated to a central system once sensor readings change or are predicted to change. However, the continuous operation of sensors drains the battery supply of mobile devices quickly. So the challenge is to derive a schedule that minimizes energy consumption while avoiding delays in detecting changes in context state.

Taleb et al. [14] presented a dynamic means of generating a sensing schedule by maximizing a cumulative reward function that accounts for the energy consumption and delay of individual sensors. Rachuri et al. [26,27] proposed an adaptive sensor triggering method that uses a feedback mechanism to decrease or increase sensor inactivity time by a multiplicative function based on the current classification of the context state. Context states are classified as either missable or unmissable using a Gaussian mixture model classifier linear reward-inaction algorithm. A missable event corresponds to no change in the state or state change that was not of interest leading to an increase in the period sensor inactivity, whereas an unmissable event corresponds to a state change of interest leading to a decrease in the period. Yurur et al. [28] presented a framework to recognize activity with a generalized expectation-maximization algorithm to achieve a trade-off between energy consumption and accuracy. The state transition probabilities of a hidden Markov model update, i.e., the sensors trigger, when an entropy rate of the user state transition matrix converges on a stable value.

In a recent approach, Janko et al. [29] used the same method introduced in [13] to select the duration after which to turn off the sensors, to match the calculated length of time spent in each context state from the steady-state condition of the Markov Chain. As such, when a context state is detected, the method turns off the sensors until the context state is expected to change. Another approach called ESGeo by Liu et al. [30] reduces energy consumption by scheduling mobile device scanning for crowdsourcing applications. The scheduling is based on a user’s historical trajectory and geographic grid information, by adapting the scanning operation in areas of probable encounter with other devices. Lastly, Tal et al. [31] determined a sampling policy dynamically for a combination of sensors by solving a convex optimization formulation combining sensor sampling costs and information loss. The sensor sampling costs are formulated in relation to energy consumption, and the information loss based on the KL-divergence between the actual context state and a latent context vector.

### 2.3. Sensor Selection and Scheduling

A few works of research aimed to combine both techniques in one framework; however, they all followed a hierarchical structure. Wang et al. [32] presented a hierarchical sensor management system called the Energy Efficient Mobile Sensing System (EEMSS) that first selects the sensors and then schedules sensing while minimizing a trade-off among accuracy, delay, and energy consumption. The framework consists of a sensor management scheme that manually links the user’s states with specific sensors by deciding which sensors to activate based on the currently recognized state. When a state transition gets detected, the next set of sensors in the sequence is activated. Moreover, a sensing schedule is manually generated through empirical tests to address the energy, accuracy, and latency trade-off. This approach does not allow for adaptability and finding the Pareto optimal trade-off. Lee et al. [33] presented a framework called Orchestrator, which is a resource coordination system that satisfies the resource demands of the multiple applications and system-wide policies, and meets the resource availability of devices. A processing planner generates multiple plans, pre-defined by developers, which specify the combinations of sensors with the associated accuracy in context recognition. Only some plans are selected based on whether they support the context recognition requests, minimize energy consumption, and maximize recognition accuracy with available resources. Furthermore, sensor triggering and data transmission are performed periodically at fixed time intervals, according to the estimated energy level availability and accuracy requirement. Sarker et al. [15] presented a hierarchical scheme for selecting and scheduling sensors. A k-means clustering algorithm is used to differentiate redundant and useful data from sensors and calculate the number of samples required to accurately detect a context. For the sensor selection mechanism, the authors suggested the use of an accelerometer by default. In the sensor scheduling stage, a multiplicative increase and multiplicative decrease (MIMD) algorithm is applied for duty-cycling each selected sensor according to the required number of samples calculated.

More recently, Mehdi et al. [34] proposed two methods to reduce energy consumption. The first is to only turn on the sensors once a monitoring watchdog sensor detects sudden changes in patient condition, and the second is a sampling rate adaptation method using chi-square test and Lagrange interpolation while maintaining accurate context recognition. Moreover, the authors in the authors in [35] expanded on the multi-objective optimization problem introduced in [13] that trades off between accuracy and energy consumption, by adding constraints to solve it with a combination of NSGA-II and constraint handling techniques. The constraints introduced to ensure the accuracy of important context states do not deviate significantly from the maximum, keep the accuracy of each context state within a certain range of one another, increase sensor operation if the accuracy is below a threshold, and use as few sensors as possible. The authors of [36] proposed to combine the methods in [13], called the setting-to-context assignment (SCA), and in [29], called the duty-cycle-to-context assignment (DCA) and the model trees (MT) method in [22]. The combined method first selects the sensors using the MT on the current context, thus each context has a different MT, then uses SCA to evaluate assigning the selected sensors to the context and uses the result as input to the DCA method to evaluate the obtained duty-cycle. The final assignment is selected based on the combination of evaluation from the SCA and DCA methods that result in minimal overall energy consumption and classification accuracy.

Taleb et al. [16] also considered decisions by both sensor selection and scheduling. The method relies on an ontology that contains specifications such as sensors and machine learning parameters. The approach filters out combinations of sensor groups that do not meet manually set accuracy and energy budget constraints. The group with the minimum energy consumption per trigger is selected. The framework uses a Viterbi algorithm [14] to generate the sensing schedules of the selected sensors. The sensing schedule based on a user state behavioral model predicts when the user may change state. The last step in the algorithm is to synchronize the schedules of multiple sensors that are common across different context recognition requests. The limitation of that approach is that it does not take into account how the operation of the chosen sensor groups will impact energy consumption and delay in state change detection. Our work proposes a holistic approach that takes into account the energy and delay to recognize multiple contexts simultaneously.

## 3. Problem Description and Formulation

**Figure 1:**
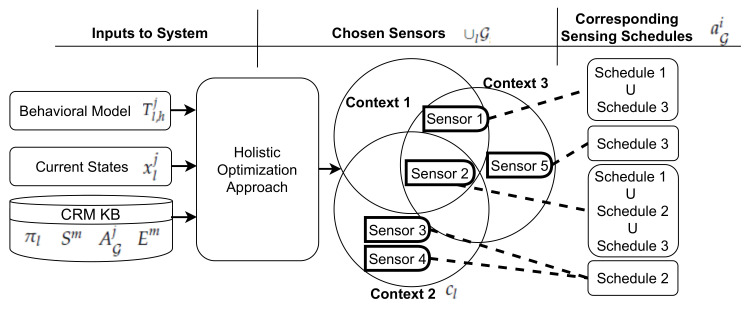
System description. The inputs are the user’s current context states, the behavior model, and a context recognition knowledge base (CRM KB). The outputs are the selected sensors and the sensing schedules for each context. The notation presented is described in Section 3.1.

The problem addressed by the holistic optimization approach is the excessive energy consumption and delay in context state change detection associated with sensor usage in multi-context recognition applications. This follows the need to select sensors to conduct requested multi-context recognition while scheduling the sensor’s operation with the aim of reducing both energy and delay. The inputs and outputs of the system are illustrated in Figure 1. As input, the approach assumes the availability of a knowledge base called a context recognition knowledge base (CRM KB) that contains information about context recognition models, the sensors they can use, and accuracies that can be achieved along with sensors’ specifications. Another input is the current state of the user as detected by the running multi-context recognition models. Last is a behavioral model that represents the user’s behavior in the recognizable states of each context, in terms of how long a user is likely to remain in a state before changing to another. The outputs of the system are the sensors selected to recognize each requested context and the sensing schedules for each sensor. Sensors that are common to multiple contexts have multiple corresponding schedules for the different context recognition models using them. The multiple sensing schedules of the common sensors are merged, as represented by the union symbol in Figure 1, through a synchronization process to take advantage of synergistic effects.

For a particular context state, sensing switches to continuous once the time interval exceeds the maximum time a user spends in a particular context state. Continuous sensing is applied because past the maximum time the user’s behavior is unpredictable, due to the lack of data. Thus, the approach cannot adapt the sensor schedule to fit the user’s behavior appropriately. Continuous sensing would certainly increase energy consumption; however, the alternative is to schedule sensor operation without any information on user behavior, which would lead to inappropriate operation of the context recognition application and delays. We assume that during continuous sensing there are minimal delays in context recognition that cannot be reduced due to the fuzzy event boundaries, as described in [37].

For the rest of this paper, we adopt the notation $c_{l}$ to represent a context with l=1,…,L, where *L* is the total number of contexts that can be recognized by the system. For each context $c_{l}$, the states are denoted by $x_{l}^{j}$, where *j* indicates the specific context state such that $j=1,\ldots ,J_{l}$, and $J_{l}$ is the number of states for context $c_{l}$. For example, the activity context can be of the following states: walking, sitting, or working. The location context can be at home, work, or a cafe. The emotion context can have the following states: happy, sad, and neutral. Each context $c_{l}$ requires sensory data accessible by the mobile and wearable devices handling the computations required for the recognition. Hereafter, when *l* appears as an index for a term. It means that that term is related to context $c_{l}$. Moreover, a table summarizing all the notation used is provided in Appendix A.

The following subsections provide a detailed description of the input requirements for our approach and the outputs. Section 3.1 describes the CRM KB by providing the design and the notation used. Section 3.2 explains the history of past user behavior or the behavior model, the sensing schedules, and their synchronization. Finally, Section 3.3 presents the mathematical formulation.

### 3.1. Knowledge Base for Context Recognition Models

**Figure 2:**
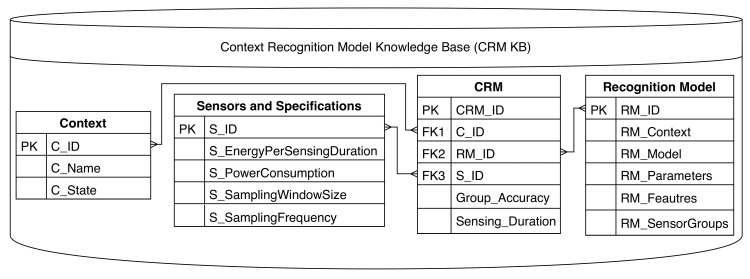
The context recognition model knowledge base (CRM KB), containing the information relevant to context recognition: (1) context; (2) sensors and specifications; (3) recognition model; (4) the associations of the three together to recognize a context.

Our solution makes use of the wealth of past knowledge in the field of context recognition stored in a context recognition models knowledge base (CRM KB). The CRM KB may be in the form of a database, as used by our approach, or an ontology, as used in [16]. For this research, the information used in the database was collected from established context recognition applications published in peer-reviewed papers in addition to sensor specification manuals made available by manufacturers. The CRM KB illustrated in Figure 2 contains:

- A context table containing the primary key, the names of contexts that may be recognized (C_Name), and the different possible context states (C_State).
- A recognition model table containing the target context for the model (RM_Context), the machine learning model (RM_Model), the parameters of the model (RM_Parameters), the features required by the model (RM_Features), and the sensors used by the model (RM_SensorGroups).
- A sensors and specifications table containing several features for each sensor: the instantaneous power consumption (S_PowerCon-sumption) used to pre-compute the energy consumption for a duration of sensor operation or trigger, the energy per sensor trigger (S_EnergyPerSensingDuration), the sampling frequency (S_SamplingFrequency), and the sampling window size (S_SamplingWindowSize).
- A context recognition model relationship table associating the models and sensors to use with each context. The table contains has links (foreign keys) to the other three entity tables. The table also shown the accuracy achieved by each combination (model, context, sensors) and the sensing duration during triggers.

The information used for solving the optimization problem is extracted for the proposed solution by querying the knowledge base. The information is presented with its respective notation, which is explained in the mathematical formulations below.

- The set of possible sensor groups for each context is denoted by $\pi_{l}$.
- Each sensor group within $\pi_{l}$ is represented by $G_{n}^{l}$, where *n* represents one of the possible groups of sensors to recognize $c_{l}$ such that $n=1,\ldots ,N_{l}$, and $N_{l}$ is the total number of groups in $\pi_{l}$. To simplify the notation, we use G in place of $G_{n}^{l}$ where possible henceforth.
- Each sensor is denoted by $S^{m}$, where *m* indicates the specific sensor. Assuming the availability of *M* embedded and wearable sensors, m=1,…,M. A sensor can belong to different sensor groups. The sensors that make up a group are typically available in mobile and wearable devices, such as a smartphone and smartwatch. Examples of sensors are a GPS, accelerometer, and a gyroscope.
- The energy consumption for each sensor group G is denoted by $E_{G}$. This energy is generated from sensor operation, CPU processing, and data transmission as in the case of a wearable sensor.
- The one-vs.-all classification percentage accuracy, specifically, the percentage of true positives achieved by each sensor group G when detecting contextual state $x_{l}^{j}$, is denoted by $A_{G}^{j}$.
- The time to recognize a state $x_{l}^{j}$ is denoted by $\delta_{G}$. $\delta_{G}$ includes the time to turn the sensors on and collect enough sensory data to recognize a context state.
- The specifications needed to recognize a particular context include: possible states $x_{l}^{j}$ in a given context $c_{l}$, ML models that can be used with a given choice of sensors, and data features collected for each ML model.

### 3.2. Time Spent in Each Context State

**Figure 3:**
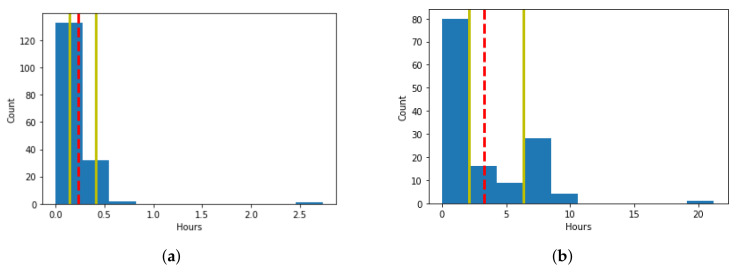
Distribution of durations spent in (**a**) “Walk” and (**b**) “Rest” states in the activity context.

Our solution makes use of the user’s past behavior to derive sensing schedules customized for particular users’ habits and behaviors in particular states. A previous approach [14] modeled user behavior by assuming a single pattern of behavior for each state represented by a single derived time limit for the user in those states. A time limit is a point in time at which the user is most likely to change their state, so the pattern of behavior modeled with the time limit is based on when the user switches context states. We propose an alternative method that makes a more realistic assumption of the user’s behavior by accounting for the variations in the user’s behavioral pattern within each state. The variations are due situation specific changes, such as the day of the week or the time of day. The previous approach would take the average of all the historic record, whereas in our approach multiple times are set as time limits to account for behavior variability. For example, the user may usually walk around 5 min every day in the morning or around 25 min some days in the evening. In other cases, the user may engage in an activity several times a day and for various periods of time, from minutes to hours, such as resting, which includes sleeping, napping, and lying down. Such variations cannot be modeled accurately by a single time limit. Accordingly, the assumption we make is that the user changes their context state at a certain time, and if not, the state will change at another time in the future. As a result, a user can have multiple time limits within each state to account for multiple patterns of behavior, instead of assuming a single pattern of behavior, as modeled by one time limit. To illustrate the difference between the resulting time limits of the two methods, Figure 3 illustrates the durations spent in the two activity states “Walk” and “Rest”, obtained from the dataset used in our experiments, with the red dashed-line representing a single time limit obtained by the method of [14], and the yellow lines representing the multiple time limits obtained by our method. As such, the multiple time limits enable our method to capture multiple behaviors of the user for the same state.

Our proposed method uses a frequent pattern mining approach to derive these multiple time limits. We capture the most frequent times spent in each state. The method requires two parameters to be specified: (1) the size of the histogram bins reflecting the desired granularity for the patterns of time limits and (2) the threshold of counts within a bin to consider the pattern frequent enough. These two parameters can be set based on the desired time resolution and what the system designer deems as frequent. For the purpose of our experimentation, a histogram of the different values with bin sizes equal to 10% of the longest duration for the state was made. The threshold of counts was selected to be 5% of the durations recorded for the state. The time limits are then computed by taking the middle duration of the bins that exceeded the threshold of counts. Additionally, if there are four or more consecutive highly populated bins, the time limit calculation method combines two consecutive bins into a single highly populated bin, which is then used to compute the time limit. This is done to avoid having too many consecutive time limits, which might impact energy consumption unnecessarily. The duration of the bin in the middle is taken as a time limit. In Figure 3b there are four highly populated bins, which are the first four that are captured by the two time limits represented by the yellow lines. The red dashed line in both figures indicates the time limit obtained by averaging all the durations.

Since the system tracks the time spent in each state, the behavioral model adapts to changes in user behavior. The model adapts by creating a new time limit, modifying an old one for a new behavior, or removing an invalid time limit, though incremental changes in recorded behavior. When the count of a bin changes around the 5% threshold, the list of time patterns or time limits for each state is updated. The updates come in the form of (1) the addition of a new time limit if the count for a bin increases above the 5% threshold, (2) the merging of two time limits if a new time limit is obtained adjacent to an old one, (3) the removal of a time limit if the count for a bin decreases below the 5% threshold, and (4) the adjustment of a time limit formed by the merging two adjacent time limits after one of the corresponding bins drops below the 5% threshold. For example, going back to Figure 3a, if the user records even more time walking under 15 min (the first bar), so that the count exceeds 95% of all the count, the time limit associated with the second bar would be dropped due to not meeting the 5% threshold.

The resulting time limits for each state $x_{l}^{j}$ are denoted by $T_{l,h}^{j}$, where the *h* index represents the time limit in ascending order, where h=1,…,H. *H* is the total number of time limits for a state and $T_{l,H}^{j}$ is the time limit with the longest duration. The time limits, $T_{l,h}^{j}$, are used to generate a sensing schedule, denoted by $a_{G}^{i}$, for a sensor group G, where a sensing decision is made every $\delta_{G}$ seconds, and the each particular time instance is referred to as $t_{G}^{i}$, where $i=1,\ldots ,I_{G}$, and $I_{G}$ is the last decision instance before sensing becomes continuous and is computed as shown in (1).

(1) $$I_{G}=\frac{T_{l,H}^{j}}{\delta_{G}}$$

We provide the following practical example to illustrate the use of sensing schedules in our approach. A user with a smartphone and smartwatch, each having a corresponding set of sensors, ran a context recognition application while at home that required samples from sensors to recognize the state of the location context $c_{1}$ and activity context $c_{3}$. After having selected the sensors to be used for multi-context recognition, the selected sensor groups recognized that the user was walking, denoted by state $x_{1}^{1}$, at home, denoted by $x_{3}^{1}$. The sensing schedule was generated in such a way as to activate the sensors most often when the user was most likely to change from a walking state to another state $x_{1}^{2}$ at the time limits $T_{3,1}^{1}$ and $T_{3,2}^{1}$, or changes location to $x_{3}^{2}$ around time limit $T_{1}^{2}$. Once the $T_{1}^{2}$ and $T_{3,2}^{1}$ passed, i.e., the last time limit of each context state, the sensors ran continuously until a new state was detected. After detecting the new state, the same process occurred again.

**Figure 4:**
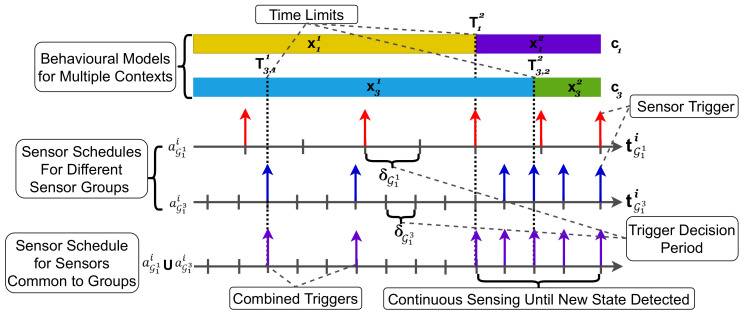
An illustration of the triggering decision interval, sensing schedules, and synchronization procedure of 2 sensor groups used to recognize different contexts while having a common sensor.

Figure 4 illustrates the two associated sensing schedules ($a_{G_{1}^{1}}^{i}$ and $a_{G_{1}^{3}}^{i}$) of the preceding example, for sensor groups ($G_{1}^{1}$ and $G_{1}^{3}$) when recognizing context $c_{1}$ and $c_{3}$, respectively. $a_{G_{1}^{1}}^{i}$ has a triggering decision period of $\delta_{G_{1}^{1}}$ and $a_{G_{1}^{3}}^{i}$ has a period of $\delta_{G_{1}^{3}}$. The user’s behavior model captures the historical patterns of the user’s behavior in different context states. These patterns are represented by time limits denoted by $T_{l,h}^{j}$ that indicate when there is a likelihood of change in a context state. On the top of Figure 4, the behavior model is shown for each context state $x_{l}^{j}$ with the corresponding time limits $T_{l,h}^{j}$. The time limits influence the triggering decisions, as seen for $a_{G_{1}^{1}}^{i}$ and $a_{G_{1}^{3}}^{i}$; sensor triggering becomes more frequent when approaching a time limit, and sparser otherwise. Moreover, if sensor groups $G_{1}^{1}$ and $G_{1}^{3}$ have common sensors, then the schedule of the common sensor is $a_{G_{1}^{1}}^{i}\cup a_{G_{1}^{3}}^{i}$, which is the synchronization of the two sensing schedules $a_{G_{1}^{1}}^{i}$ and $a_{G_{1}^{3}}^{i}$, as illustrated as the bottom sensing schedule in Figure 4. The synchronization process creates a single sensing schedule formed as a union of different schedules and is applied to the common sensors in the corresponding sensor groups. Moreover, if there are sensor triggers that are relatively close, e.g., $\delta_{G}$ seconds apart or less, then they are combined into one trigger in the synchronized schedule. Examples for the combination of proximate triggers are shown in Figure 4 for the first two triggers in the synchronized schedule at the bottom.

### 3.3. Mathematical Formulation

Mathematically, we formulate the optimization problem as a weighted sum of two objectives, minimizing energy consumption and delay. The formulation has two additional constraints. The first family of constraints ensures that a selected sensor must have the needed energy to operate. In other words, a sensor’s required energy ($E^{m}$) is less than the sensor’s energy budget ($E_{B}^{m}$). The second family of constraints ensures that not more than one group of sensors can be used to recognize a specific context at any time. The specific choices of sensor groups and their schedules are obtained by aiming to achieve an optimal trade-off between energy and delay. In this section, we use G in place of G to simplify the notation and to highlight the more relevant concepts.

The formulation is expressed in terms of the combination of energy consumption of the union of sensor groups $E_{\cup_{l}G}^{j}$ and cumulative delays for all contexts in contextual state detection $D_{\cup_{l}G}^{j}$. The energy and delay components in the sum are normalized by their respective maximum possible values of energy $E_{\cup_{l}G,\max}^{j}$ and delay $D_{l,\max}^{j}$. The terms are weighted by a weighting factor $\omega_{l}$ multiplied by the boolean decision variable $y_{G}$, which has a value of either 1 or 0. The boolean decision variable represents the system’s choice of sensor group to recognize its associated context; thus, multiplying by $y_{G}$ ensures that only the sensor groups being considered to have their associated energy and delay values factored into the objective function value to enable proper comparison of different sensor group combinations.

(2) $$\min_{y_{G},\;a_{S^{m}}^{i}}\sum_{G}\left(\omega_{l}\frac{E_{\cup_{l}G}^{j}}{E_{\cup_{l}G,\max}}+\left(1-\omega_{l}\right)\frac{D_{\cup_{l}G}^{j}}{D_{l,\max}^{j}}\right)\left(\prod_{l}y_{G}\right)$$

with the following constraints:

(3) $$E^{m}\leq E_{B}^{m},\;S^{m}\in \cup_{l}G\prod_{l}y_{G}\;\forall \;c_{l}$$

(4) $$\sum_{n}y_{G}\leq 1,\;\forall \;c_{l}$$

- $E_{\cup_{l}G}^{j}$ is the total energy consumption resulting from triggering the union of sensors $\cup_{l}G$. $E_{\cup_{l}G}^{j}$ is computed as the count of sensor triggers, where triggers are the sensor activations with all associated operations, multiplied by the energy consumption per trigger $E_{\cup_{l}G}$ for the sensor groups recognizing each context state according to the respective sensing schedules. Since the user does not always spend the same amount of time in each context state, the energy term is averaged over multiple scenarios of times spent by a user in a particular context.
- $D_{\cup_{l}G}^{j}$ represents the delays incurred in using the selected sensors for context recognition of the different states $x_{l}^{j}$. $D_{\cup_{l}G}^{j}$ is computed as the difference in time between when the state changes and the sensor trigger following the change. Just like the energy term, the delay term is also averaged over multiple scenarios.
- $y_{G}$ is a boolean decision variable denoting the selection of sensor group G. $y_{G}$ is 1 when the groups of sensors are being considered in the computations; otherwise, $y_{G}$ is 0 when the sensor group’s energy is not included in the minimization term.
- $a_{S^{m}}^{i}$ is a vector of boolean decision variables denoting the sensing schedule for the sensors belonging to the selected combination of sensor groups $S^{m}\in \cup_{l}G\prod_{l}y_{G}$ at different instances $t_{G}^{i}$ as follows:
  (5) $$a_{S^{m}}^{i}=\left\{\begin{array}{c} 1,\mathrm{when}\;\mathrm{sensor}S^{m}\mathrm{is}\;\mathrm{triggered}\;\mathrm{at}t_{G}^{i}\\ 0,\mathrm{otherwise} \end{array}\right.$$
- $\omega_{l}$ is a weighting factor that provides a balance between the two objectives and is user-specified depending on the application. For example, in the health context delay in state change might be more important than the energy consumption; thus, it should have a smaller value of $\omega_{l}$.
- $E_{\cup_{l}G,\max}^{j}$ is the maximum energy consumption value, which is equal to the largest time limit $T_{l,H}^{j}$ multiplied by $\Omega_{\cup_{l}G}$, where $\Omega_{\cup_{l}G}$ is the power consumption value of the union of sensor groups $\cup_{l}G$ when the sensors are operating continuously.
  (6) $$E_{\cup_{l}G,\max}^{j}=\Omega_{\cup_{l}G}\times T_{l,H}^{j}$$
- $D_{l,\max}^{j}$ is the maximum delay value, which is equivalent to the largest time limit $T_{l,H}^{j}$ since it represents the case when the generated sensing schedule does not decide to sense until $t_{G}^{i}=T_{l,H}^{j}$.
  (7) $$D_{l,\max}^{j}=T_{l,H}^{j}$$
- $E_{B}^{m}$ is the energy budget value, which is equivalent to the energy available in the relevant power source of the sensors belonging to the selected combination of sensor groups; i.e., $S^{m}\in \cup_{l}G\prod_{l}y_{G}$. The sensors’ power source could be a smartphone or smartwatch battery, or a dedicated rechargeable battery.

Equation (2) exploits the synergy between the selected groups of sensors by considering the total energy cost consumed by the union of sensors $\cup_{l}G$ of the selected groups to recognize the different contexts. Constraint (3) enforces an energy budget $E_{B}^{m}$, specific to each sensor $S^{m}$ belonging to the candidate sensor groups, on the sensor of the candidate sensor groups. This is to ensure that the system can only choose sensors that have not depleted their energy supplies. If for a specific context the energy required by a group of sensors to recognize a context exceeds the energy budget, then an alternative group is selected. If there are no alternatives, then the recognition of that particular context is discontinued. Constraint (4) states that exactly one group of sensors for each context is to be chosen. The objective function (2) takes the form of a mixed-integer linear programming (MILP) problem, where the one variable is a boolean variable for the choice of sensor groups and the other is a vector of boolean variables of varying size representing the sensing schedule of a selected sensor. The solution is guaranteed to converge to a global minimum because of the convexity of linear problems [38]. The optimization problem is solved by choosing sensors to recognize the requested context with the respective sequence of sensing decisions for each sensor that minimizes the objective function (2).

## 4. Overview of the Proposed System

**Figure 5:**
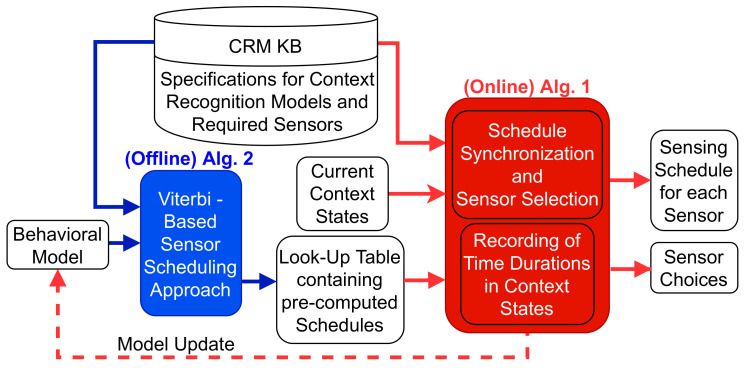
The proposed holistic optimization approach is split into two stages: an online stage (red) which determines the best combinations of sensors and their sensing schedules obtained directly from the LUT according to Algorithm 1, and an offline stage (blue) which provides the best sensing schedule for a particular context state and a group of sensors and stores them in a look-up table (LUT) according to Algorithm 2.

Here we present our solution to the problem described in the previous section. The solution to the optimization problem needs to be used in real-time by context recognition systems to decide on the groups of sensors and when to trigger them to consume the least energy and achieve minimal delay in multi-context recognition. To achieve real-time performance, we propose to pre-compute optimized sensing schedules and recognition delays for different possible combinations of sensor groups and context states and make decisions among these choices online. As a result, the approach is split into two stages, an online stage and an offline stage, as illustrated in Figure 5.

The offline stage addresses the problem of determining the optimized sensing schedule that provides the best trade-off between energy consumption and delay for each particular context. These combinations are then stored in a look-up-table (LUT) that is used online. The entries in the LUT have the form [<context state, group of sensors> : sensing schedule]. The online stage addresses the problem of finding the groups of sensors and their schedules to achieve the best synergy between sensor options for the simultaneous recognition of multiple contexts. The online system provides a multi-context trade-off between energy and delay.

The following subsections provide detailed descriptions of the two components of our approach. Section 4.1 describes the online system which selects the sensors and synchronizes the sensing schedules obtained from the LUT. Section 4.2 describes the offline system, which pre-computes the sensing schedules and stores them in the LUT, and is further decomposed into three sub-subsections, which further detail parts of the Viterbi based algorithm.

### 4.1. Online System

To select the groups of sensors and their schedules, the system takes in as inputs in the online stage (1) the current context states of the user, (2) a knowledge base for context recognition models (CRM KB) with the models’ accuracy and sensor specifications, and (3) a look-up-table (LUT) produced by the Viterbi offline system capturing pre-computed optimized sensing schedules for each combination of context state and related group of sensors. The outputs of the online system are their choices of sensors for the current context states and their sensing schedules. A sensing schedule is composed of a sequence of sensing decisions that directs the sensor operations during the recognition of a particular context state. The system also records the actual times spent by the user in particular context states. These times are fed back to the offline system for updates to the user’s behavioral model.

Algorithm 1, found on the following page, details the procedure for the online system that runs every time a context state changes, i.e., when the sensors’ latest measurements lead to a different inference than the state that was inferred from the previous measurements. The approach starts by computing combinations of sensor groups $\cup_{l}G$ from the set of sensor groups $\pi_{l}$ capable of recognizing the desired contexts. For each possible sensor group combination $\cup_{l}G$, the sensing schedules are extracted from the LUT for each sensor group G. The sensing schedules are then synchronized for sensors that are common across multiple sensor groups.
**Algorithm 1** Online algorithm for sensor selection with sensing schedules.**Inputs:**$x_{l}^{j}$, $\pi_{l}$, $S^{m}$, $E^{m}$, $E_{G}^{j}$, $D_{G}^{j}$$a_{G}^{i}$ $x_{l}^{j}$: the current context state of the user contexts ∀l=1,2,…,L  **From CRM KB:** $\pi_{l}$: the set of possible sensor groups G for each of the requested *L* contexts $S^{m}$: the available sensors $E^{m}$: the energy consumption by each sensor $S^{m}$  **From LUT:** $E_{G}^{j}$: the pre-computed energy consumption for each group of sensors G in recognizing context state $x_{l}^{j}$ $D_{G}^{j}$: the pre-computed delay of each sensor group G in recognizing context state $x_{l}^{j}$ $a_{G}^{i}$: the pre-computed optimized sensing schedules for the sensor groups in $\pi_{l}$ in recognizing each context state.**Output:**$y_{G}$, $a_{G}^{i}$  $y_{G}$: The boolean variable representing the selected sensor groups to recognize multiple contexts states $x_{l}^{j}$  $a_{S^{m}}^{i}$: Sensing schedules corresponding to the sensors of the selected group including the synchronized schedules1:Derive all the possible union of sensor groups $\cup_{l}G$ available in $\pi_{l}$2:**for** each $\cup_{l}G$ **do**3:  **for** each $S^{m}\in \cup_{l}G$ **do**4:    **if** $S^{m}\in G$ **then**5:     Assign $a_{G}^{i}$ obtained from the LUT to $S^{m}$ of group G as $a_{S^{m}}^{i}$6:    **else if** $S^{m}\in G_{w}^{l}$ & $G_{q}^{l}$ for w≠q **then**7:     Synchronize sensing schedules $a_{G_{w}^{l}}^{i}$ & $a_{G_{q}^{l}}^{i}$ and assign to $S^{m}$ as $a_{S^{m}}^{i}$8:    **end if**9:  **end for**10:  Calculate objective function values according to equation (2)11:  Track sensors $S^{m}\in \cup_{l}G$ and sensing schedules $a_{S^{m}}^{i}$ with minimum objective function value according to equation (2)12:**end for**13:Return sensors $S^{m}\in \cup_{l}G$ with $y_{G}=1$ and the corresponding sensing schedules $a_{S^{m}}^{i}$ having the minimum objective value14:**if** state $x_{l}^{j}$ changes to $\bar{x_{l}^{j}}$ **then**15:  Record time spent in changed context state $x_{l}^{j}$16:**end if**

For each unique sensor $S^{m}$ in G, the LUT sensing schedule is assigned to it $a_{S^{m}}^{i}=a_{G}^{i}$. For the common sensors, the schedules are synchronized by taking the union of the schedules. Using the schedule for every sensor, the energy and delay values are computed to determine the objective function of Equation (2). For sensors that are unique to a context, the energy is computed based on the sensing schedule extracted from the LUT without additional modifications. For sensors that are common, the energy is computed based on the synchronized union of sensing schedules from different contexts. Finally, the combination of sensor groups with the minimum objective function is selected. When a new context state is detected, the time spent in the previous context state is fed back to the offline system for updating the behavior model.

For the experiments, we chose the value of the weighting parameter $\omega_{l}$ described in Equation (2) as $\omega_{l}=0.5$. We provided the energy and delay terms equal weight. However, the user may choose to have a different value for $\omega_{l}$ depending on the priority of energy reduction versus delay.

An example of how to calculate the objective function for a given group of sensors with their sensing schedule is given in Appendix B.

### 4.2. Offline System: Viterbi-Based Sensing Schedules

To generate the sensing schedules in the offline stage, the Viterbi algorithm is applied to each combination of context state and sensor group. The process takes into account the user’s behavioral model, which captures the likelihood of changing to a new context state. The schedules and the associated energy consumption and delay values are stored in the LUT that is used in the online stage. An optimized sensing schedule balances energy and delay, since triggering the sensors too often leads to excessive energy consumption, and too seldom leads to increased delays. We propose to reformulate the optimization as a Viterbi formulation [39], with the aim of maximizing the utility of the possible combinations of sensors in recognizing multiple contexts concurrently. A key factor in Viterbi formulation is the definition of a reward function for the target problem.

In determining an efficient reward function and deriving an optimized sensing schedule there are two aspects that are useful. The first aspect is knowing at any time the likelihood of state change, called state survival probability. The survival probability would be high when it is unlikely that the user will have a state change. On the other hand, the probability would be low when it is imminent that the user will change state. For example, if a user is likely to spend a lot of time in a particular state, there would be no need to trigger the sensors often until the state is expected to change. The second aspect is the times spent by a user in each state. The users may have different habits, resulting in different possible times spent in given states. These times, called time limits, help in assessing the state survival probabilities. The largest time limit for each state determines when the system should switch to continuous sensing.

Section 4.2.1 describes the survival probability, which represents the likely hood of remaining in a context state at any time, in an illustrative manner. Appendix C provides the corresponding mathematical representations. Section 4.2.2 describes a reward function used by the Viterbi algorithm to determine sensing decisions at each decision period that form the sensing schedules.

#### 4.2.1. User Behavior State Survival Probability

Different patterns of behavior may be followed by each user for each context state, which has to be accounted for by the sensing schedule. The same functions apply to both single time limits and multiple time limits, but here we show the functions for a single time limit for simplicity. The influence of the time limits on the behavior model is represented by the survival probability of the user remaining in the state, $p^{j}\left(t_{G},T_{l,h}^{j}\right)$. The likelihood of staying in the state correlates with the length of time *t* the user spends in the state. When the duration reaches the time limit $T_{l,h}^{j}$, the survival probability reaches a near-zero value, where a state change is expected with a probability of nearly 1. We used a value close to zero, denoted by ε, for implementation purposes. During the time preceding the time limits, the behavior of the survival probability is subject to the distribution of the durations found in the statistical record for each state. For example, if the user is less likely to change state at any time, the survival probability may be modeled as a linear or exponential function. Exponential decay in the survival probability would reflect a faster increase in the probability of state change.

**Figure 6:**
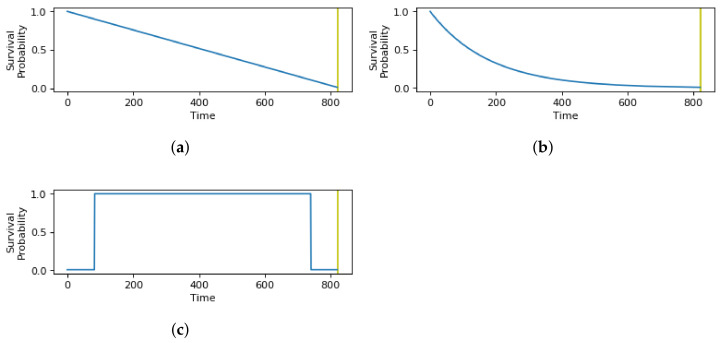
Illustration of the survival probability using the (**a**) linear function, (**b**) exponential function, (**c**) uniform function.

Figure 6a illustrates an example of the survival probability with a linear decay rate, while Figure 6b illustrates an example of the survival probability with an exponential decay rate. Another example of survival probability may be represented by a constant for the case when the user has an equal probability of changing to a new state independent of when the state changed. As an example, the user may have only one particular time limit for a state. Once the user is in that state, they will not change state until the time limit elapses. Figure 6c illustrates an example of the survival probability with a constant or uniform representation.

**Figure 7:**
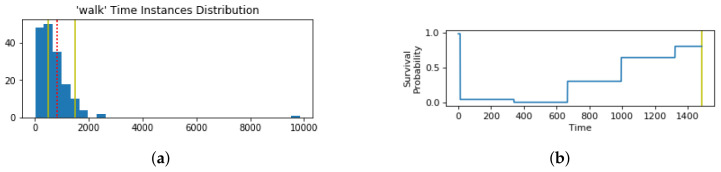
(**a**) Histogram of duration spent in a state found in the user historical record; (**b**) survival probability using the distribution function for the survival probability.

Another way to derive the survival probability is by first creating a histogram of the user’s behavior in every state, then taking the compliment of the density of each bin in the histogram as the value of the survival probability at the time corresponding to each bin. Figure 7a shows the distribution of durations for the “walk” activity with the corresponding time limits, and the resulting survival probability is illustrated in Figure 7b. We discuss the mathematical representation of the different possible survival probabilities in the following section. Their impacts are further evaluated in Section 5.2.4, and the corresponding mathematical notation is provided in Appendix C.

#### 4.2.2. Reward Function

To generate a sensing schedule, the Viterbi based algorithm makes decisions at each time instant $t_{G}^{i}$: whether to trigger the sensors or not to trigger, based on a reward metric. The possible sequential decisions form a path of decision nodes of sensing or non-sensing nodes, linked by edges arriving at the final node at $t_{G}^{i}=T_{l,H}^{j}$, where the user is most likely to transition into another context state. The utility of transition between nodes, or the sensor triggering decision, is measured by a metric called reward function R(), with each edge having its own defined metric. The Viterbi algorithm aims at finding the sensing schedule decisions that maximize the accumulated reward function over the entire schedule. Each sensing decision has an associated value and maximizes the accumulated reward results by consuming less energy and incurring less delay. Mathematically, the Viterbi objective function aims to find the sequence of triggering decisions $a_{G}^{i}$ that maximizes the accumulated reward function as follows:

(8) $$\underset{a_{G}^{i}}{\mathrm{argmax}}\sum_{i=1}^{I_{G}}R\left(a_{G}^{i},\Delta t_{G}^{i+1},A_{G}^{j},p^{j}\left(t_{G}^{i},T_{l,h}^{j}\right)\right)$$

- $a_{G}^{i}$ represents the sensing schedule, where index *i* represents the sensor triggering decision at time $t_{G}^{i}$.
- $I_{G}$ is the last decision instance before sensing becomes continuous, where $I_{G}=\frac{T_{l,H}^{j}}{\delta_{G}}$.
- $\Delta t_{G}^{i+1}$ is the time elapsed since the preceding sensor “Sense” decision.
- $A_{G}^{j}$ the accuracy achieved by sensor group G in detecting contextual state $x_{l}^{j}$
- $p^{j}\left(t_{G}^{i},T_{l,h}^{j}\right)$ is the state survival probability.

We propose a reward function that takes into account the energy consumption of the sensors relative to each other and the accuracy of the sensor group in recognizing context state. The reward function also accounts for accuracy, since an incorrectly recognized context state would lead to additional delays due to the time that would elapse until the state is correctly recognized. An incorrect recognition, either a false positive or a false negative, is not explicitly identified by the system, so the delays caused by incorrect detection are not precisely measured. To deal with this type of delay, the reward function prompts sensing to increase when the accuracy is low to minimize reduce delay attributed to misclassification and to decrease when the accuracy is high to reduce energy consumption.

The function also takes into account the possible delay, based on the time period since the preceding sensor activation decision, i.e., when the sensing schedule last turned the sensor on. The state of the user is not known before triggering the sensors, so the reward function is probabilistic and depends on the likelihood of having transitioned into a new state, which is captured by the survival probability $p^{j}\left(t_{G}^{i},T_{l,h}^{j}\right)$. For $t_{G}^{i}=T_{l,h}^{j}$, the survival probability reduces to near zero, i.e., ε, and sensing switches to continuous.

As a result, the value of the reward function depends on the combination of the decision taken and the survival probability value. The reward function is divided into two instantaneous rewards r(). The first instantaneous reward represents the case where a state does not change at the new time instance, and the second instantaneous reward is when a state change occurs. Mathematically, the reward function can be derived from the expected value of the instantaneous rewards as follows:

(9) $$\begin{array}{c} R\left(a_{G}^{i},\Delta t_{G}^{i+1},A_{G}^{j},p^{j}\left(t_{G}^{i},T_{l,h}^{j}\right)\right)=E_{s}\left[r\left(x_{l}^{j},a_{G}^{i+1},\Delta t_{G}^{i+1},A_{G}^{j}\right)\right]\\ \;=p^{j}\left(t_{G}^{i},T_{l,h}^{j}\right)\cdot r\left(x_{l}^{j},a_{G}^{i+1},\Delta t_{G}^{i+1},A_{G}^{j}\right)+\left(1-p^{j}\left(t_{G}^{i},T_{l,h}^{j}\right)\right)\cdot r\left(\bar{x_{l}^{j}},a_{G}^{i+1},\Delta t_{G}^{i+1},A_{G}^{j}\right) \end{array}$$

$E_{s}$ is the expected reward that depends on whether the current state being recognized has changed or not, i.e., $x_{l}^{j}$ or $\bar{x_{l}^{j}}$, respectively. At each time instant $t_{G}^{i}$ there is a corresponding probability $p^{j}\left(t_{G}^{i},T_{l,H}^{j}\right)$. To make decisions at each step on whether to trigger the sensors or not, a value is attributed to the instantaneous reward r(), to calculate the best triggering decision for the next step, i.e., $a_{G}^{i+1}$. To penalize long periods of inactivity of the sensors that might lead to delays, and to reward quick recognition of a state change, we measure the difference in time between the next decision and the last triggering instance as $\Delta t_{G}^{i+1}$.

The instantaneous reward reflects the impact of a sensing decision on consumed energy and delay in detecting a state change. The energy reward component is computed as follows:

(10) $$-\frac{E_{G}^{j}}{E_{G,\max}}$$

where $E_{G}$ is the energy cost of triggering sensor group G, and it is normalized by the maximum instantaneous energy value $E_{G,\max}$ of the available sensor groups to recognize the requested context. The delay component is reflected in the combination of two terms: the probability of recognition captured by the accuracy of the model $A_{G}$ and the amount of delay captured by time elapsed $\Delta t_{G}^{i+1}$ since the last sensing decision as follows:

(11) $$-\alpha .\left(\Delta t_{G}^{i+1}\right)+\beta .\frac{A_{G}}{\left(\Delta t_{G}^{i+1}\right)}$$

**Figure 8:**
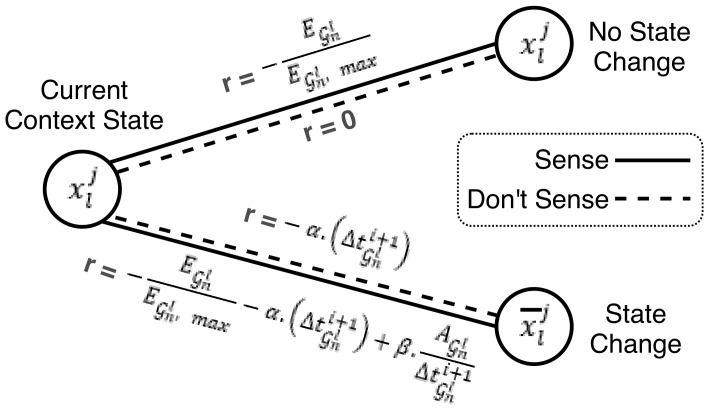
State transition diagram showing the instantaneous reward values for the different conditions of “Sense” and “Do not Sense” sensor triggering decision, depending on whether the state has changed or not.

Depending on the combination of triggering decisions made (“Sense” or “Do not Sense”) and user state $x_{l}^{j}$ or $\bar{x_{l}^{j}}$ (“No State Change” or “State Change”), the instantaneous reward is a weighted sum of the two components: (1) energy and (2) time elapsed. Figure 8 shows the instantaneous reward values with the state change to triggering action conditions. The triggering decision is represented in the figure by a line for the “Sense” decision and a dashed line for the “Do not Sense” decision.

When the decision is to sense and the state does not change ($x_{l}^{j}$), then the instantaneous reward is represented by the energy cost penalty. However, if the state has indeed changed ($\bar{x_{l}^{j}}$), then in addition to the energy penalty, the time elapsed component is added. The time elapsed component is composed of two terms, a delay penalty weighted by α, and a state recognition reward weighted by β. The delay term penalizes the instantaneous reward in proportion to the time elapsed $\Delta t_{G}^{i+1}$. The state recognition term is inversely proportional to $\Delta t_{G}^{i+1}$ to reward recognizing the change in state as soon as possible and is proportional to the accuracy of the sensor group $A_{G}^{j}$ in recognizing state $x_{l}^{j}$ to account for the chance of incorrect recognition of the context state. α and β are weighting factors, and their combination is specified by finding the optimal Pareto solution to an optimization formulation (12), as applied to a single sensor group takes the form:

(12) $$\underset{\left(\alpha ,\beta \right)}{\mathrm{argmin}}\;\omega_{l}\frac{E_{G}^{j}}{E_{G,\max}^{j}}+\left(1-\omega_{l}\right)\frac{D_{G}^{j}}{D_{l,\max}^{j}}$$

The terms in the formulation are the same as those found in Equation (2), only they are related to a single sensor group ($E_{G}^{j}$), rather than the union of multiple groups ($\cup_{l}E_{G}^{j}$). Once the optimal combination of α and β is obtained, they are applied to the reward function to find the optimal sensing schedule for each sensor group, following the steps described in Algorithm 2, found on the following page, to fill the LUT with the generated sensing schedules.
**Algorithm 2** Offline algorithm for sensor scheduling.**Input:**$\pi_{l}$, $A_{G}$, $E^{m}$, $T_{l,h}^{j}$  **From CRM KB:** $\pi_{l}$: the choices of groups of sensors G for each of the requested *L* contexts $A_{G}$: the accuracy of group G $E^{m}$: the energy consumption by each sensor $S^{m}$  **From User Behavior:** $T_{l,h}^{j}$: the time limits of the context states $x_{l}^{j}$**Output:**$a_{G}^{i}$, $E_{G}^{j}$, $D_{G}^{j}$ $a_{G}^{i}$: the sensing schedules for the sensor groups in $\pi_{l}$ recognizing each context. $E_{G}^{j}$: the energy consumption for each group of sensors G recognizing context state $x_{l}^{j}$ $D_{G}^{j}$: the delay of each sensor group G in recognizing context state $x_{l}^{j}$1:**for** each sensor group $G\in \pi_{l}$ **do**2:  Calculate $E_{G}$ as the sum of $E^{m}$ of $S^{m}\in G$3:  **for** each context state $x_{l}^{j}$ **do**4:   **for** each (α,β) pair **do**5:    Run Viterbi algorithm to determine sensing schedule for G in recognizing state $x_{l}^{j}$6:    Compute the objective function value according to (12)7:    Track the resulting energy consumption, delay, and the (α,β) pair for the derived sensing schedule.8:   **end for**9:   Store the sensing schedule with the minimum objective function value according to (12) and the associated energy consumption ($E_{G}^{j}$), delay ($D_{G}^{j}$), and (α,β) pair in the Look-Up Table (LUT)10:  **end for**11:**end for**

## 5. Experiments and Results

We conducted a set of experiments to evaluate the performance of the proposed method. The experiments included the evaluation of three aspects: the holistic approach that simultaneously determines the optimized group of sensors and their schedules, the behavior model, and the survival probability. The holistic approach was compared to the state-of-the-art [16], which used a single pattern of time spent in a given state and was based on a hierarchical approach—selecting the groups and then determining the sensing schedules. As such, in the following subsections we provide the setup and system parameters used in the experiments (Section 5.1); the results of the experiments, including the evaluations of the three aspects, in addition to corresponding performance analysis (Section 5.2); and lastly, the computational complexity of our approach (Section 5.3).

### 5.1. Experimental Setup

#### 5.1.1. Dataset

The sensors’ specifications used in the experiments are listed in Table 1, along with the related references. The accuracy values obtained from the references are for the best-case outcomes. To reflect a practicals scenario of using relatively accurate recognition models, we selected the sensor groups that achieved recognition accuracy above 70%. The information found in the CRM KB was obtained from published research papers on energy-efficient context recognition systems. In the experiments, we conducted we used the information found in [40,41,42,43,44,45].

To simulate context scenarios, we generated a dataset using a third-party application called “Smarter Time” that ran on a real device and tracked time spent on activities of daily life, location, and fitness/health. The application initially requires the user to self-report context states until enough sensor data are collected, allowing the application’s context recognition system to automatically recognize the context states. The collection period was two months for a volunteer, following the user’s daily behavior without intrusion. Three categories of context were recorded: “Activity”, “Location”, and “Health”. The tracked states for the activity context included walking, eating, driving, reading, resting, etc.; and the monitored locations were home, work, gym, shop, parents, friends, etc. For the experiments, we focused on a subset of the available context states. Activity states were “Walk,” “Sit,” and “Jog”. Location states included “Home” and “Work”. Health states included “Healthy” and “Unhealthy”.

At any time a user could be in any combination of states in the monitored contexts. For example, a multi-context scenario may have consisted of the case where the user was sitting at home and was healthy. The user would have been in the “Sit” state for activity context, the “Home” state for location context, and the “Healthy” for state health context. The frequent time limits representing the user’s behavior in the different states were calculated as described in Section 3.2 and are summarized in Table 2. If a state was not recognized, it remained unlabeled until it was labeled by the user. All unlabeled data were placed in a distinct context state labeled “No Category”, and so the approach operates with an unlabeled state as it would with a recognized state.

#### 5.1.2. System Parameters

**Figure 9:**
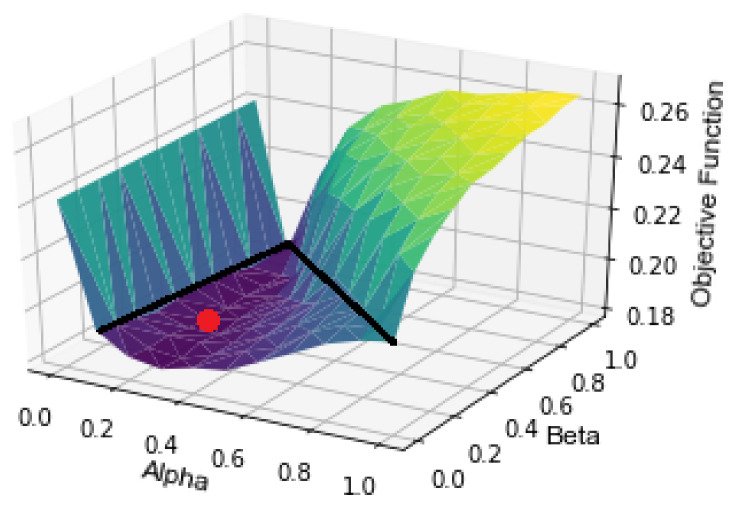
Sample plot of the parameters alpha and beta vs. the resulting objective value, resulting from the sensing schedules for different combinations of (α,β).

The Pareto optimal (α,β) combination was selected by enumerating the different possible combinations to find the pair that gave the minimum point according to Equation (12). As shown in Figure 9, it was observed that values of α+β>1 led to a sudden increase in objective function and divergence from the global optimal. Furthermore, the choice of α=0 led to no sensing, which is to be avoided. As a result, the values of (α,β) were limited to satisfy the condition:

(13) α+β≤1&α>0

**Figure 10:**
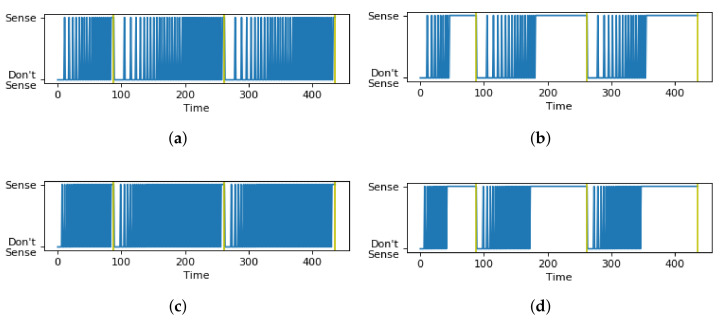
Sensing schedules for (α,β) combinations showing the continuous sensor triggering phenomenon for values of (**a**) α=0.3, β=0.7; (**b**) α=0.3, β=0.8; (**c**) α=0.8, β=0.2; and (**d**) α=0.8, β=0.3.

In Figure 9, the red dot represents the chosen optimal combination (α,β) and the black lines represent a boundary condition of (α,β) beyond which the objective function value increases exponentially, and the values of (α,β) beyond the boundary were not tested. In the sensing schedules in Figure 10a,c, where values of (α,β) are within the bounds of search space, the sensing schedule alternates between “Sense” and “Do not Sense” up to each time limit. On the other hand, the sensing schedules in Figure 10b,d, where (α,β) are outside the bounds of the search space, show a consistent “Sense” decision without alternating back to “Do not Sense” long before reaching the time limits. For the experiments, we set δ=10 s for all contexts to simplify the experiments. Moreover, the exponential function for the survival probability was used for both the holistic and hierarchical approaches.

### 5.2. A Comparison to a Prior Hierarchical Approach

In this section, we compare our proposed holistic approach to sensor selection and scheduling from prior work (EGO [16]) based on a hierarchical decision that first selects sensors then decides on their schedule. We considered all possible combinations of states for the three monitored contexts detailed in Table 2, making up 12 context scenarios.

#### 5.2.1. Effect of Holistic Approach Versus Hierarchical

**Figure 11:**
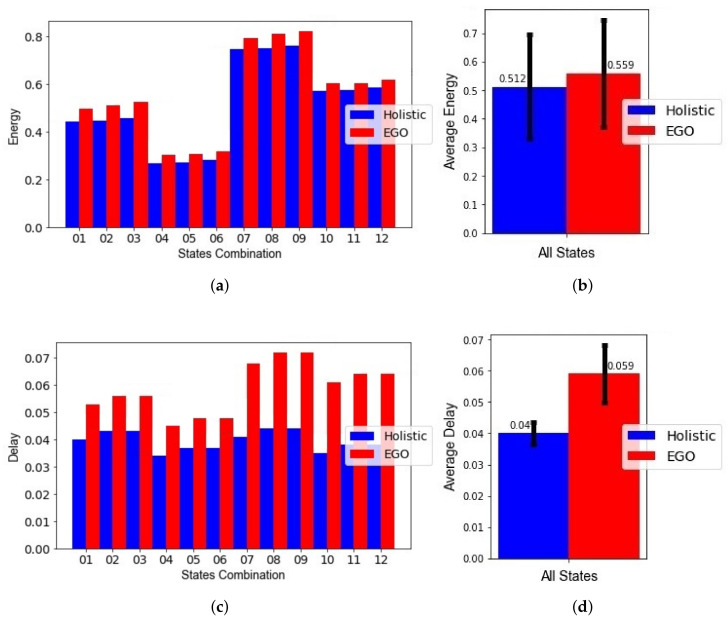
A comparison between EGO and the holistic approach both using the new behavioral model for (**a**) normalized energy and (**c**) normalized delay for each state’s combination scenario; (**b**) average normalized energy and (**d**) average normalized delay for all states’ combination scenarios.

To test the merit of the holistic versus the hierarchical approach without other improvements, we assumed all other conditions were the same for both approaches. This included using the same sensor information and the same behavioral model for both. In particular, we used the proposed behavior model of multiple time limits for both methods. The results of the experiments are shown in Figure 11.

For each of the 12 context scenarios, Figure 11a shows the normalized energy values obtained for both holistic and hierarchical approach (EGO). Figure 11c shows the normalized delay values. For all the cases, the holistic approach performed better than the hierarchical in both delay and energy consumption. The actual energy consumption values in Joules and delay in seconds can be obtained by multiplying the normalized values with the maximum values, as previously illustrated in computations of energy and delay. In Figure 11b, the holistic approach resulted in a normalized energy value of 0.512 (104 mJ), and the average normalized energy value for EGO was 0.559 (132 mJ), meaning the holistic approach showed an average improvement of 8.4% in normalized energy. In Figure 11d, the holistic approach resulted in a normalized delay value of 0.04 (15 s), while the average delay value for EGO was 0.059 (20 s), meaning the holistic approach showed an average improvement of 32% in normalized delay. When summing the delay and energy, the cumulative improvement was 10.7% in the objective function. Additionally, the standard deviation of the normalized energy value for the holistic approach was smaller than the standard deviation of EGO, as can be seen by the black line at the center of the bars in Figure 11b, indicating a more stable performance.

#### 5.2.2. Performance Analysis

**Figure 12:**
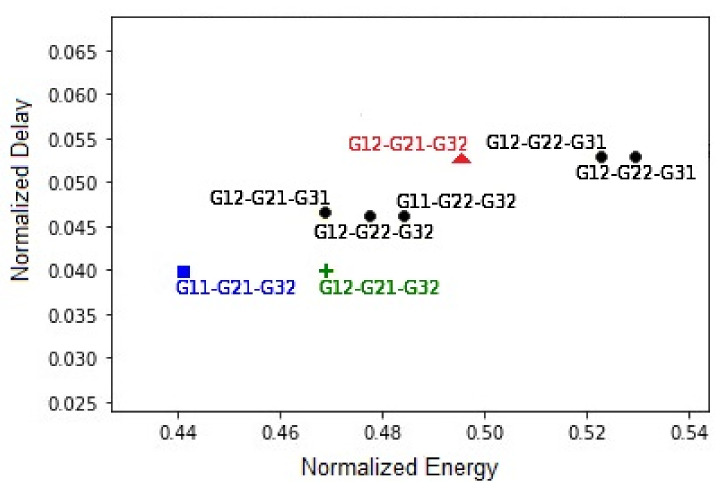
Representations of all sensor group combinations with their respective normalized delay and energy values.

To study more closely the effect of using the holistic approach, we examined one of the context scenarios to find out why the holistic approach performed better: the first state combination, where the states were, “Jog,” “Work,” and “Unhealthy,” i.e., the scenario for the bar in Figure 11a labeled “01.” As such, we looked at all the possible sensor group combinations with their respective normalized energy and normalized delay values, as shown in Figure 12, which represents all the sensor group combinations, with their respective normalized delay and energy values. The black dots are for sensor combinations that were not selected by either method, the blue square is for the group combination selected by the holistic approach, and the red triangle is for the groups selected by EGO [16]. Additionally, the figure shows the sensor group combination chosen by EGO, but from the application of the Viterbi algorithm used in the holistic approach represented by the green cross.

The groups selected by the holistic approach were $G_{1}^{1}$ (G11), $G_{1}^{2}$ (G21), and $G_{2}^{3}$ (G32); the groups selected by EGO were $G_{2}^{1}$ (G12), $G_{1}^{2}$ (G21), and $G_{2}^{3}$ (G32). Both methods selected the same groups, $G_{1}^{2}$ and $G_{2}^{3}$, for the location and health contexts, but different groups, $G_{1}^{1}$ and $G_{2}^{1}$, for the activity context for the holistic approach and EGO, respectively. The combination selected by the holistic approach has a lower objective function value (0.48), whereas the combination selected by EGO has a greater value (0.55). As reflected in Table 1, when considering the combinations selected by the holistic approach, group $G_{1}^{1}$ had the accelerometer sensor, with energy consumption per trigger of 0.3 mJ, in common with group $G_{1}^{2}$, and the total instantaneous energy consumption was 1.2 mJ. When considering the combinations selected by EGO, $G_{1}^{1}$ had Bluetooth Low Energy (BLE), with energy consumption per trigger of being 0.1 mJ, in common with group $G_{1}^{2}$; and the total instantaneous energy consumption was 0.85 mJ. Although the total instantaneous energy consumption for the group selected by EGO was less than the holistic approach’s choice, the energy consumption per trigger of the common sensor was different, 0.3 mJ for the accelerometer and 0.1 mJ for the BLE. Thus, after synchronizing the sensing schedules for the common sensors, $a_{G_{1}^{1}}^{i}$ and $a_{G_{1}^{2}}^{i}$ for the accelerometer and $a_{G_{2}^{1}}^{i}$ and $a_{G_{1}^{2}}^{i}$ for the BLE, the total energy consumption of the combination selected by the holistic approach results fell behind the overall energy consumption of the combination selected by EGO.

**Figure 13:**
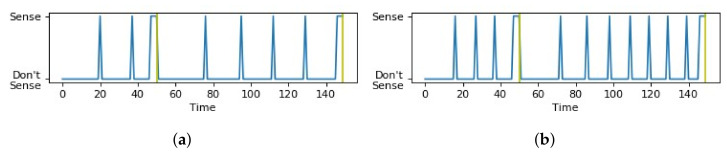
Sensing schedule generated using the Viterbi algorithm for sensor group $G_{2}^{3}$ with the reward functions of (**a**) EGO and (**b**) the holistic approach.

In addition to energy improvements, Figure 12 shows an improvement in delay. The improvement is attributed to improved representation of the energy consumption of the sensor groups in the instantaneous reward of the Viterbi algorithm. In EGO the energy is represented in terms of a constant and does not account for the differences in energy consumption between possible selections of sensor groups to recognize a context. Figure 13 illustrates the difference in the sensing schedules between EGO, Figure 13a, and the holistic approach, Figure 13b, for the same selection sensor group $G_{2}^{3}$. More frequent sensor triggering led to more energy consumption but a lower delay value. The increased energy consumption was compensated by the selection of additional common sensors, leading to reduced energy consumption overall the expected operation time.

#### 5.2.3. Impact of Multiple Time Limits in the Behavioral Model

**Figure 14:**
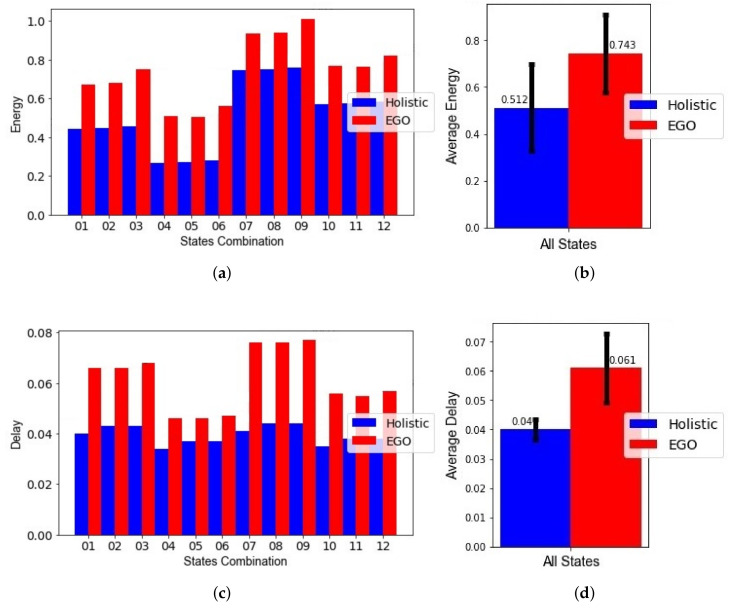
A comparison between the holistic approach using the new behavioral model and EGO with its own behavior model for (**a**) normalized energy and (**c**) normalized delay for each state’s combination scenario; (**b**) average normalized energy and (**d**) average normalized delay for all states’ combination scenarios.

Here we compare the proposed solution with both aspects of holistic optimization and the improved behavioral model against the original state-of-the-art (EGO) using their own behavioral model, which averages the historical duration data spent in each state and adds a fraction of the standard deviation to compute a single time limit per state. The normalized energy, normalized delay, and resulting objective function values for the 12 combinations of states and the average values are examined for the holistic approach and EGO in Figure 14. To keep the comparison fair, the maximum values in the denominators used for normalization were kept the same across both approaches, because the maximum values of energy and delay were different for the two approaches due to the difference in behavior modeling.

**Figure 15:**
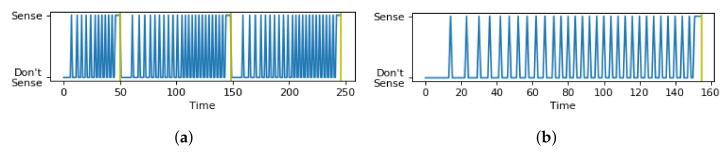
Sensing schedules using the same sensor group to detect the same state, (**a**) using the binning technique or (**b**) using the VCAMS [14] method for modeling the behavior of the user.

The use of the different modeling techniques, i.e., the binning technique and the averaging technique, resulted in different sensing schedules applied to a sensor group when detecting a state. To illustrate the difference, Figure 15a has three time limits, obtained using the binning technique. Each time a time limit is approached, more sensing is encouraged by the Viterbi algorithm, but once it is passed, sensing triggers become scarcer. Figure 15b shows the result of the previous method in the literature [14] that relies on only one time limit. Note that at the last time limit $T_{l,H}^{j}$, continuous sensing is applied until a change in state is detected.

For each of the 12 context scenarios, Figure 14a, shows the normalized energy values obtained for both holistic and hierarchical approaches (EGO). Figure 14c shows the normalized delay values. As before, the holistic approach performed better than the hierarchical in both delay in state change detection and energy consumption for all the cases. As shown in Figure 14b, the holistic approach resulted in an average reduction of 31.1% in normalized energy. As shown in Figure 14d, the holistic approach showed an average reduction of 34.4% in normalized delay, which is the same as before, meaning the modified behavioral model reduces energy consumption while maintaining the same levels of delay. Taking the sum of the energy and delay terms, the holistic approach showed an average reduction of 31.3% for the objective function value using the holistic approach compared to EGO. In summary, the improvements attributed to the use of the binning technique are 22% for the normalized energy and 2.4% for the normalized delay.

#### 5.2.4. Impact of Survival Probability

**Figure 16:**
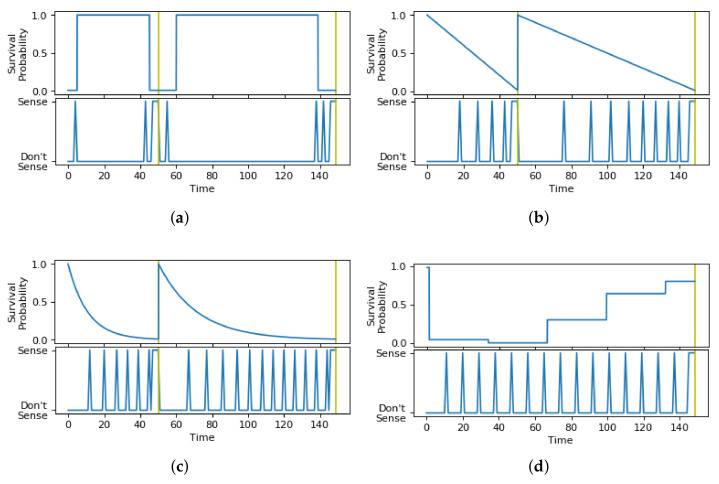
Illustration of impacts on the sensing schedule generated by the Viterbi algorithm using the (**a**) uniform function, (**b**) linear function, (**c**) exponential function, (**d**) distribution function.

Finally, we tested the impact of the survival probability function on the sensing schedule generated by the Viterbi algorithm, as illustrated in Figure 16. The uniform function in Figure 16a shows sensing decisions at the start of the schedule and around the time limits. The uniform function may lead to optimized results in cases of high confidence in which the user only changes their state near the obtained time limits, as that would reduce the delays obtained while reducing energy consumption drastically. For this experiment, the uniform function performed poorly because the user’s data are more stochastic and less determinable; i.e., we could not know for sure that the state of the user will change exactly according to the behavioral model. Therefore, the distribution of the duration data needs to be taken into consideration when choosing the function modeling the survival probability of context states. The linear function in Figure 16b shows sensing decisions in the schedule that gradually become more frequent until reaching the time limits. The exponential function in Figure 16c shows sensing decisions in the schedule that quickly become more frequent, faster than that of the linear function, until reaching the time limits. The distribution function in Figure 16d shows sensing decisions in the schedule of a constant frequency, starting at the lowest point in the survival probability.

**Figure 17:**
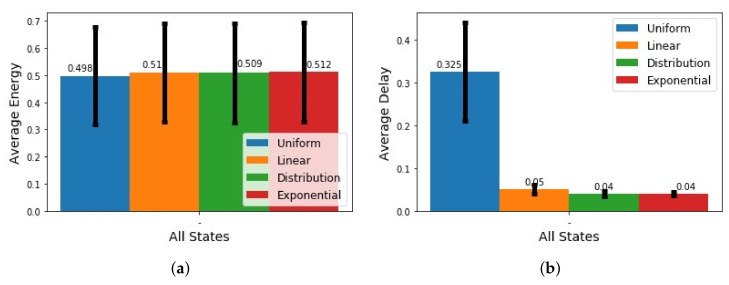
(**a**) Average normalized energy consumption values for all context scenarios using the different survival probabilities: (**b**) average normalized delay values for all context scenarios using the different survival probabilities.

The results for all context scenarios of the average normalized energy consumption and average normalized delay are shown in Figure 17a,b, respectively. In Figure 17a, the best result in terms of energy consumption is the uniform function, followed by the distribution function, and the worst is the exponential function, followed by the linear function. In Figure 17b, the best results in terms of delay are for the exponential and the distribution functions, and the worst is the uniform function. From these results, it appears that having the sensing schedules formed of a periodic triggering, as is the case when the distribution function is used, gives the best trade-off between energy consumption and state change detection delay. However, that might not always be the case, as it would depend on the circumstances of the context recognition application and user behavior. Thus, the choice of survival probability function will impact the overall outcome, so it is beneficial to have an accurate representation of state survival probability to ensure optimized sensing decisions.

### 5.3. Computational Complexity

In the offline stage, the holistic Viterbi algorithm is applied repeatedly to find the Pareto optimal weighting parameters. The number of times the algorithm is applied depends on how many combinations of (α,β) are to be examined; i.e., complexity is $O(N^{2})$. We note that because of the (α,β) boundary conditions, as opposed to the previous work in VCAMS [14], the complexity becomes $O(\frac{1}{2}N^{2})$. Additionally, since each iteration of (α,β) combination is independent of the others, the process of finding the optimal combination is parallelizable. The complexity of applying the Viterbi algorithm is $O(I.A^{2})$, where *I* is the total number of instances in which triggering decisions are made, and A is the number of distinct actions that can possibly be taken which are to sense or not to sense. As for the complexity in the online stage, the computational complexity is dominated by the number of possible combinations $\Pi_{l=1}^{L}G^{{}^{\prime }}$ for sensor groups to recognize the requested contexts. Thus, the complexity is O(L.N), where *N* is the number of groups per context and *L* is the number of requested contexts.

## 6. Procedural Guide and Discussion

We present here a guide for practitioners to follow when implementing our approach and discuss the feasibility of it. To properly implement our approach first requires the collection and ordering of two sets of information. The first set of information is that which is found in the CRM knowledge base; the second set is the data collected from the user’s historic behavior in different context states, which could in be the context of activity, location, health, body-posture, emotion, etc. In order to feasibly define the CRM knowledge base, there are two credible sources: industry provided sensor specification manuals and peer-reviewed research papers of context recognition models. Sensor specification manuals provide the information required by our approach about sensor power consumption and the range of sampling frequency. The sensor’s power consumption may be derived from the manual’s voltage or current information, in combination with the voltage or current information of the respective power supply, which the sampling frequency range is usually directly given. It is also worth noting that our approach may use virtual sensors as well. By definition, a virtual sensor is an emulation of a sensor that obtains its measurements from a physical sensor and can be used for context recognition to take advantage of its sensor-fusion capabilities [46,47]. Such virtual sensors can be used by our approach as long as the required information can be collected.

On the other hand, peer-reviewed papers provide the information required by our approach about the context recognition models, such as the required sensor group, one-vs.-all classification accuracy, duration needed to recognize a state otherwise known as the sampling window size, the states that can be detected by the context recognition model, and the model’s associated features, parameters, and implementations. The accuracy is determined by the designers of the context recognition application during their experiments evaluating their methods using the training/testing/validation methodology. The sampling window size is either given directly or can be derived as the number of samples required to calculate a feature divided by the minimum sampling frequency. Moreover, some approaches use the overlap between sampling windows; in such cases, the sampling window should be at least two sampling periods. The context states and group of sensors used are usually listed in the paper, or in the associated datasets. As for implementations of the context recognition models, they are provided online by the authors, can be requested from the authors, or recreated based on the information about the models found in the associated peer-reviewed papers, such as the required features, parameters, and architecture. A general CRM knowledge base would contain the information extracted from a plethora of context recognition models, and this is valid as similar approaches, such as the state-of-the-art hierarchical approach presented in EGO [16], define a knowledge base in the form of an ontology. Accordingly, a personalized on-device CRM knowledge base would be derived from the general CRM knowledge base for each user based on the available sensors and devices.

The second set of information, the user’s historic behavior in different context states, is collected by monitoring the duration spent by the user in each context state. The monitoring process implies some initial manual specification, by the user of the windows of time spent in context states, as sensor readings are collected throughout his/her daily life. For instance, the method followed by Vaizman et al. [48], who developed a mobile application deployed on smartphones to collect in-the-wild data via sensor measurements, requires past and near-future self-reporting of combinations of relevant context-labels. The application captures user contexts state durations, which can be used to represent user behavior, as confirmed by [49]. For example, after getting up from rest, the user indicates that the duration spent in that state, which the application does not yet recognize is in fact the “Rest” state, and afterward the application automatically recognizes whenever the user is in the rest state. The self-reporting of labels is done only in the cases where the context recognition models are still unable to directly recognize the context states of the user. For example, a new location may be detected, but the system does not know what the state of the location context is, whether it is home or work, which needs to be defined by the user. Most of the states are registered within the first week, as in the data collection method followed by [48], which lasted for 1 week. In some cases, there are states that do not show up during the first week. For example, the user might visit a new location after the first week; however, this location may be visited consistently, so it should be classified. Thus, the approach cannot accurately personalize to users without first collecting enough information about their historic behavior. In fact, the monitoring period is related to an issue known in the industry as the cold start problem, which is faced by, for example, recommender systems [50]. This issue can be overcome by utilizing one of the two following methods:

- A personalized configuration setup to obtain a rough estimate of the user’s behavior model to be used initially until more accurate data have been collected.
- A generalized configuration setup obtained from a pool of users; for example, it may average the time limits of different users, or it may use the most common pattern of behavior found across users.

The configuration setup would prompt the user to enter information about the time spent in states, giving multiple entry options, to generate the time limits, in addition to prompts that inquire about the user’s current state in the requested contexts. These solutions would allow the system to start immediately and then slowly becoming more accurate as more information is gathered.

Finally, to conclude the guide and to better understand the scope of our approach, we reiterate our assumptions: (1) during continuous sensing, there are minimal delays in context recognition which cannot be reduced due to the fuzzy event boundaries, as described in [37]; (2) the availability of a knowledge base that contains information about context recognition models; and (3) a user has multiple behavioral patterns associated with context states which may be taken advantage of by our approach. Moreover, the limitations of our approach are (1) the inability to detect incorrect context recognition and (2) absence of accuracy as an objective of the multi-objective optimization formula. To address the first limitation, an ensemble decision technique may be applied that takes into account multiple sensor readings to decide on the states of the recognized contexts. As for the second limitation, a multi-objective optimization problem may be formulated to include accuracy by adding an error term and thus maintain the minimization form.

## 7. Conclusions

This paper described a holistic optimization approach to minimize energy consumption and delays in the simultaneous detection of multiple context states. The problem was formulated as an optimization problem to decide on the sensors and their sensing schedules simultaneously. The contributions included a new user behavioral model based on capturing the user’s frequent patterns in every context state and the Viterbi instantaneous reward functions captured normalized energies allowing comparison across groups of sensors and the accuracy in context recognition as it impacts delays. Compared to the previous state-of-the-art, the proposed solution showed an improvement of 31% in energy reduction and was 34% faster in state change detection. We were able to reduce the number of computations needed to find the optimal parameters for the Viterbi algorithm. Moreover, we showed the adaptation of the method to different state survival probabilities and the importance of accurately representing the user’s behavior. For future work, the problem can be extended to find the group of sensors that can not only trade off energy and delay but also achieve a balance with the best accuracy in multi-context recognition.

## Figures and Tables

**Table 1:** CRM KB sensor groups’ data.

Context	Ref.	(G)	Sensors ($S^{m}$)	($E_{G}$) (mJ)	($A_{G}$)
Activity	[40]	$G_{1}^{1}$	Accelerometer, Gyroscope, Microphone	0.55	83%
	[41]	$G_{2}^{1}$	Accelerometer, Bluetooth Low Energy	0.4	89%
Location	[42]	$G_{1}^{2}$	Bluetooth Low Energy, WiFi	0.35	89%
	[43]	$G_{2}^{2}$	Accelerometer, Proximity, Light, GSM, Magnetometer	0.84	78%
Health	[44]	$G_{1}^{3}$	Accelerometer, Electrocardiogram, Skin Temperature	0.62	91%
	[45]	$G_{2}^{3}$	Electrocardiogram, Electrodermal Activity	0.2	92%

**Table 2:** User behavior time limits for different context states.

Context	State ($x_{l}^{j}$)	Single Time Limit ($T_{l}^{j}$)	Multiple Time Limits ($T_{l,h}^{j}$)
Activity	$x_{1}^{1}=$ “Walk”	$T_{1}^{1}=2675$	$T_{1,1}^{1}=3431$, $T_{1,2}^{1}=10274$
	$x_{1}^{2}=$ “Sit”	$T_{1}^{2}=612$	$T_{1,1}^{2}=696$, $T_{1,2}^{2}=2065$, $T_{1,3}^{2}=3433$
	$x_{1}^{3}=$“Jog”	$T_{1}^{3}=825$	$T_{1,1}^{3}=880$, $T_{1,2}^{3}=2619$, $T_{1,3}^{3}=4357$
Location	$x_{2}^{1}=$ “Home”	$T_{2}^{1}=1929$	$T_{2,1}^{1}=1720$, $T_{2,2}^{1}=1481$, $T_{2,3}^{1}=5142$
	$x_{2}^{2}=$ “Work”	$T_{2}^{2}=1548$	$T_{2,1}^{2}=503$, $T_{2,2}^{2}=1481$, $T_{2,3}^{2}=2460$
Health	$x_{3}^{1}=$ “Healthy”	$T_{3}^{1}=2334$	$T_{3,1}^{1}=2295$
	$x_{3}^{2}=$ “Unhealthy”	$T_{3}^{2}=403$	$T_{3,1}^{2}=521$

## Data Availability

The data and code used for this paper can be found here: https://github.com/RuslanKain/optimization-multi-context-recognition (accessed on 1 October 2021).

## Appendix A

On the next page is a table containing all the notation used in our approach.

**Table A1:** Table of notation.

Groups’	Notation	Descriptions
Sets	*L*	Set of contexts
	*M*	Set of available sensors $S^{m}$
	$N_{l}$	Set of sensor groups capable of recognizing context *l*
	$J_{l}$	Set of total states in context $c_{l}$
	*H*	Set of time limits in state $x_{l}^{j}$
	$\pi_{l}$	Set of sensor groups
Parameters	$c_{l}$	Particular context out of *L* total requested contexts
	$x_{l}^{j}$	Particular state in context $c_{l}$
	$G_{n}^{l}$	Set of sensor groups that can detect context $c_{l}$, where *n* corresponds to each sensor group, such that $n=1,\ldots ,N_{l}$
	$\Omega_{\cup_{l}G}$	Power consumption for continuous sensing of a union of multiple sensor groups $\cup_{l}G$ over requested contexts
	$S^{m}$	Sensor indexed by *m*
	$\delta_{G}$	Time duration to recognize a state $x_{l}^{j}$ by sensor group G
	$E_{B}^{m}$	Energy budget of sensor $S^{m}$
Optimization Values	$\omega_{l}$	Energy and delay weighting factor, where $0<\omega_{l}<1$
	$E^{m}$	Energy consumption by sensor $S^{m}$
	$E_{\cup_{l}G}^{j}$	Energy consumption by the union of multiple sensor groups $\cup_{l}G$ as operated by each group’s sensing schedule
	$A_{G}^{j}$	One-vs.-all accuracy of group G in recognizing state $x_{l}^{j}$
	$D_{\cup_{l}G}^{j}$	Delay recognizing state $x_{l}^{j}$ using the union of multiple sensor groups $\cup_{l}G$ as operated by each group’s sensing schedule
Decision Variables	$y_{G}$	Binary variable variable representing selection of group G
	$a_{G}^{i}$	Sensing schedule as a vector of boolean decision variables denoting the trigger decisions for group G: 1 for “Sense” and 0 for “Do not Sense”
	$a_{S^{m}}^{i}$	Sensing schedule of sensor $S^{m}$
Variables	$t_{G}^{i}$	Time instants related to sensor group G
	$T_{l,h}^{j}$	Time limit representing the most frequent time a user spends in-state $x_{l}^{j}$, where *h* is the number of time limits such that h=1,…,H and $T_{l,H}^{j}$ is the last time limit
	$I_{G}$	Last decision instance before sensing becomes continuous, computed as $I_{G}=\frac{T_{l,H}^{j}}{\delta_{G}}$
	$D_{l,\max}^{j}$	Maximum delay value equal to the largest time limit $T_{l,H}^{j}$
	$E_{\cup_{l}G}^{j}$	Maximum energy consumption value equal to $T_{l,H}^{j}$ by $\Omega_{\cup_{l}G}$

## Appendix B

To illustrate how we calculate the objective function for a given group of sensors with their sensing schedule, we give an example using the data shown in Table A2 showing three hypothetical scenarios of recognizing an activity state “Walk”. The context state is recognized using an accelerometer sensor that forms the group $G_{2}^{1}$, which consumes $E_{G_{2}^{1}}=0.3$ mJ of energy per trigger over $\delta_{G_{2}^{1}}=10$ s. The hypothetical scenarios include only a single context and sensor, rather than multiple, to simplify the presentation and focus on the calculation steps. However, the calculations can be easily expanded to multiple contexts by summing the objective function values for each sensor group recognizing a context, as described in the optimization formula of Equation (2).

**Table A2:** Three scenarios in “Walk” state detection with time limits of $T_{1,1}^{1}=3431$ and $T_{1,2}^{1}=10,274$ s.

Walk Activity Scenarios	Time of State Change	Trigger Time Nearest to State Change	Count of Sensor Triggers	Time to Recognize a State
(1)	2000	1990, 2020	40	10
(2)	5000	4980, 5050	90	10
(3)	12,000	10,274 ${}^{1}$	350	10

${}^{1}$ Maximum time limit.

We assume that the user has the habit of spending either $T_{1,1}^{1}=3431$ or $T_{1,2}^{1}=$ 10,274 s in this particular context. Once the time elapsed since the context state was first detected exceeds $T_{1,2}^{1}$, sensing switches to continuous. $T_{1,1}^{1}$ does not cause the sensors to operate continuously. However, $T_{1,1}^{1}$ does affect the sensing schedule, i.e., when the sensor is triggered and how often. The scenarios were selected to show the cases where (1) the state changes before $T_{1,1}^{1}$, (2) the state changes between $T_{1,1}^{1}$ and $T_{1,2}^{1}$, and (3) the state changes after $T_{1,2}^{1}$. Three scenarios are presented, capturing the energy, delay, and objective function values by taking the average values over the three scenarios and then normalizing by the maximum possible values. We note that past the time instance equivalent to the time limit $T_{1,2}^{1}$, for example, at time interval $t_{G_{2}^{1}}^{i}=$ 12,000 s, we assumed there was no delay in detection of context state change because continuous sensing was applied.

$$\begin{array}{c} \mathrm{Normalized}\;\mathrm{Energy}=\frac{E_{G}^{j}}{E_{G,\max}^{j}}=\frac{1}{3}\cdot \sum_{Scenario=1}^{3}\frac{E_{G_{2}^{1}}^{1}}{T_{1,2}^{1}\cdot E_{G_{2}^{1}}/\delta_{G_{2}^{1}}}=\frac{1}{3}\cdot \sum_{1}^{3}\frac{\#\;\mathrm{of}\;\mathrm{Triggers}\times E_{G_{2}^{1}}}{T_{1,2}^{1}\cdot E_{G_{2}^{1}}/\delta_{G_{2}^{1}}}\\ =\frac{1}{3}\cdot \frac{40\times 0.3+90\times 0.3+350\times 0.3}{10,274\times 0.3/10}≅0.47 \end{array}$$

$$\begin{array}{c} \mathrm{Normalized}\;\mathrm{Delay}=\frac{D_{G}^{j}}{D_{l,\max}^{j}}=\frac{1}{3}\cdot \sum_{Scenario=1}^{3}\frac{D_{G_{2}^{l}}^{1}}{T_{1,2}^{1}}\\ =\frac{1}{3}\cdot \sum_{1}^{3}\frac{(\mathrm{Trigger}\;\mathrm{After}\;\mathrm{Change}-\mathrm{Trigger}\;\mathrm{Before}\;\mathrm{Change})}{T_{1,2}^{1}}\\ =\frac{1}{3}\cdot \frac{(2020-1990)+(5050-4980)+0}{10274}≅0.01 \end{array}$$

$$\mathrm{Objective}\;\mathrm{Value}=\omega_{l}\frac{E_{G}^{j}}{E_{G,\max}^{j}}+\left(1-\omega_{l}\right)\frac{D_{G}^{j}}{D_{l,\max}^{j}}=0.47+0.01=0.48$$

## Appendix C

To compute the survival probability at any time during the context recognition application, we provide the equations used to compute the values. The state survival probability typically decays until a time limit is reached, then the survival probability is reset to 1. Note that just before the state change, the survival probability is assumed to reach a near-zero value. When the maximum time limit is surpassed, sensing switches to continuous; i.e., the survival probability remains near zero (ε), and we do not have information about the behavior of the user. The exponential function survival probability is described mathematically as following:

(A1) $$p^{j}\left(t_{G}^{i},T_{l,h}^{j}\right)=e^{\ln\left(\varepsilon \right).\frac{t_{G}^{i}}{T_{l,h}^{j}}}$$

An example of the constant uniform function can be described as follows: Anytime $t_{G}^{i}$ is not near a time limit, the value is set to a constant, which we represent as 1. When the time $t_{G}^{i}$ reaches a time limit or is within a fraction η of a time limit, the survival probability is set to ε. The uniform function (constant function) is described mathematically as follows:

(A2) $$p^{j}\left(t_{G}^{i},T_{l,h}^{j}\right)=\left\{\begin{array}{cc} 1, & \;\mathrm{if}\;\eta \times T_{l,h}^{j}<t_{G}^{i}<\left(1-\eta \right)\times T_{l,h}^{j}\\ \varepsilon , & \;\mathrm{otherwise}\; \end{array}\right.$$

The linear function is a linearly decaying survival probability, starting at 1 and reducing to ε when a time limit is reached, and resetting to 1 if the time limit reached is not the final time limit $T_{l,H}^{j}$, following the below equation:

(A3) $$p^{j}\left(t_{G}^{i},T_{l,h}^{j}\right)=\left(\frac{-1+\varepsilon }{T_{l,h}^{j}-T_{l,h-1}^{j}}\right)*t_{G}^{i}+1$$

The survival probability can be derived from the distribution of durations to produce what we call the distribution function. The function does not require all the time limits; it only requires the last time limit $T_{l,H}^{j}$ as follows:

1. Sort in increasing order the durations in the historical record for a given state.
2. Group the durations into 30 bins, with each bin representing 3.3% of the longest duration in the historical record.
3. Count the number of durations per bin.
4. Calculate the survival probability assigned to each bin range, using the following formulation:
  (A4) $$p^{j}\left(t_{G}^{i},T_{l,H}^{j}\right)=1-\frac{\#\;\mathrm{of}\;\mathrm{occurrences}}{\mathrm{Largest}\;\#\mathrm{of}\;\mathrm{occurrences}\forall \;\mathrm{bins}}$$
  where $t_{G}^{i}\in$ is the bin range. Moreover, we tested multiple bin sizes, 1%, 2%, 3.3%, 5%, 6.6%, and 10% of the longest duration in the historical record, with 3.3% corresponding to 30 bins, giving the best result.
